# From the parents’ perspective: a user-satisfaction survey of immunization services in Guatemala

**DOI:** 10.1186/1471-2458-14-231

**Published:** 2014-03-06

**Authors:** Lissette Barrera, Silas Pierson Trumbo, Pamela Bravo-Alcántara, Martha Velandia-González, M Carolina Danovaro-Holliday

**Affiliations:** 1 National Immunization Program, 5a. Avenida 11-40 zona 11, Colonia El Progreso, Ciudad de Guatemala, Guatemala; 2 Vanderbilt School of Medicine, Nashville, Tennessee, USA; 3 Comprehensive Family Immunization Project, Pan American Health Organization, 525 23rd Street NW, Washington, DC 20037, USA

**Keywords:** Immunization programs, Guatemala, Immunization services, User-satisfaction survey, Vaccination surveys

## Abstract

**Background:**

Immunization coverage levels in Guatemala have increased over the last two decades, but national targets of ≥95% have yet to be reached. To determine factors related to undervaccination, Guatemala’s National Immunization Program conducted a user-satisfaction survey of parents and guardians of children aged 0–5 years. Variables evaluated included parental immunization attitudes, preferences, and practices; the impact of immunization campaigns and marketing strategies; and factors inhibiting immunization.

**Methods:**

Based on administrative coverage levels and socio-demographic indicators in Guatemala’s 22 geographical departments, five were designated as low-coverage and five as high-coverage areas. Overall, 1194 parents and guardians of children aged 0–5 years were interviewed in these 10 departments. We compared indicators between low- and high-coverage areas and identified risk factors associated with undervaccination.

**Results:**

Of the 1593 children studied, 29 (1.8%) were determined to be unvaccinated, 458 (28.8%) undervaccinated, and 1106 (69.4%) fully vaccinated. In low-coverage areas, children of less educated (no education: RR = 1.49, p = 0.01; primary or less: 1.39, p = 0.009), older (aged >39 years: RR =1.31, p = 0.05), and single (RR = 1.32, p = 0.03) parents were more likely to have incomplete vaccination schedules. Similarly, factors associated with undervaccination in high-coverage areas included the caregiver’s lack of education (none: RR = 1.72, p = 0.0007; primary or less: RR = 1.30, p = 0.05) and single marital status (RR = 1.36, p = 0.03), as well as the child’s birth order (second: RR = 1.68, p = 0.003). Although users generally approved of immunization services, problems in service quality were identified. According to participants, topics such as the risk of adverse events (47.4%) and next vaccination appointments (32.3%) were inconsistently communicated to parents. Additionally, 179 (15.0%) participants reported the inability to vaccinate their child on at least one occasion. Compared to high-coverage areas, participants in low-coverage areas reported poorer service, longer wait times, and greater distances to health centers. In high-coverage areas, participants reported less knowledge about the availability of services.

**Conclusions:**

Generally, immunization barriers in Guatemala are related to problems in accessing and attaining high-quality immunization services rather than to a population that does not adequately value vaccination. We provide recommendations to aid the country in maintaining its achievements and addressing new challenges.

## Background

In the last two decades, the National Immunization Program (NIP) of Guatemala has made remarkable progress. The country was certified free of polio in 1994, with the rest of the Americas, and concluded the documentation and verification of measles, rubella, and congenital rubella syndrome (CRS) elimination in 2011 [1]. National vaccination coverage rates have also increased. Reported third-dose coverage of diphtheria, tetanus, and pertussis vaccine (DPT3) was 85% in 2011, compared to 81% in 2000 and 66% in 1990 [2,3].

Despite these achievements, coverage rates continue to fall short of national targets of ≥95% [3]. In 2011, first-dose coverage of measles-mumps-rubella containing vaccine (MMR1) was 88% and third-dose coverage of oral polio vaccine (OPV3) was 84% [3]. Moreover, coverage rates are unequal. In 2012, 25% of districts reported DPT3 coverage <80% [3]. In light of these results and the limited understanding of the causes of undervaccination in Guatemala, we conducted a user-satisfaction survey of the country’s immunization program.

Established in 1978, Guatemala’s NIP operates under the Ministry of Health and Social Protection (MSPAS in Spanish). Health units in MSPAS freely provide the majority of vaccination services, while the private sector, non-governmental organizations, and the Guatemalan Social Security Institute (IGSS in Spanish) offer supplemental services [4]. In 2011, the national immunization schedule contained antigens against 12 diseases: severe forms of tuberculosis (BCG), diphtheria, pertussis, tetanus, Hepatitis B, *Haemophilus influenzae* type b (pentavalent vaccine), poliomyelitis, rotavirus, measles, mumps, rubella (MMR), and seasonal influenza. Health workers, women of childbearing age (15–49 years), and the elderly are also vaccinated [5]. To complement routine services, health units at the local and subnational levels provide door-to-door immunization services and send vaccination brigades to low-coverage areas. The last international evaluation of the NIP was conducted in 2001, underscoring the need for an updated review of the immunization program.

In this paper, we assess parental attitudes, preferences, and practices related to vaccination in Guatemala; parental awareness of immunization campaigns and communication strategies; and barriers to immunization in the country’s health centers. We then propose interventions to raise coverage rates and identify areas where additional research is needed. Lastly, we identify lessons learned that are relevant to ongoing efforts by the Pan American Health Organization (PAHO) to develop a standardized tool for detecting missed opportunities for vaccination in the Americas.

## Methods

### Sampling

Participants in this study were required to be parents or guardians of children aged 0–5 years. Individuals employed by healthcare organizations or public relations companies were excluded.

**Figure 1:**
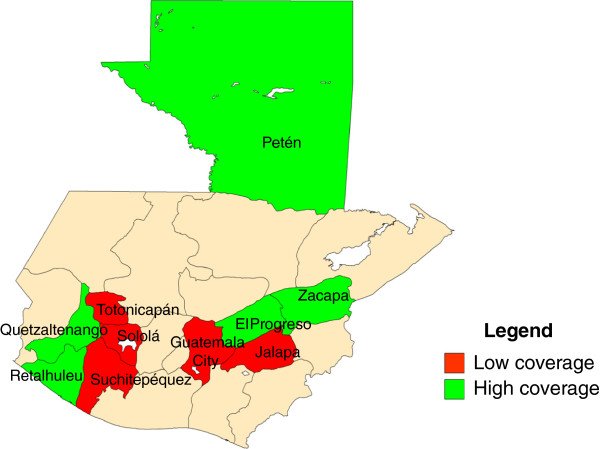
High- and low coverage departments of Guatemala, May 2011.

A multi-stage clustered sampling design was used to select participants. We classified Guatemala’s 22 geographic departments as high- or low-coverage areas based on administrative coverage rates, poverty indicators, and results from the 2008–2009 Survey on Infant and Maternal Health [4]. Of the 22 departments, we selected five low-coverage and five high-coverage areas as the survey strata. Low-coverage departments included Guatemala City, Jalapa, Sololá, Suchitepéquez, and Totonicapán; high-coverage departments included El Progreso, Petén, Quetzaltenango, Retalhuleu, and Zacapa (Figure 1). According to data from Guatemala’s National Institute of Statistics (INE in Spanish), the 10 departments selected represent nearly half of the country’s population [6].

Using INE’s classification system for population units, municipalities in each department were stratified into primary sampling units (PSUs) with a design effect of two. In some cases, a PSU consisted of one municipality; in others, a municipality was divided into multiple PSUs. For each PSU, the INE provided population estimates by age and gender. Overall, we identified 3426 PSUs in low-coverage departments and 2670 in high-coverage departments. PSUs were selected in each stratum with probability proportional to estimated size (PPES). A total of 84 PSUs were chosen, 42 for each type of coverage area. In each PSU, blocks and neighborhoods were designated as sample spaces (SS). Criteria for designating spaces included proximity to health centers and cities in urban areas and proximity to villages and major farms in rural areas. As with the PSUs, sample samples corresponded to the INE’s criteria for population units, and no sample samples were excluded from the study. Using PPES, we randomly selected one sample space per PSU. Within each SS, an initial home was randomly selected and subsequent homes were chosen in a clockwise direction until 12–15 interviews had been conducted.

While we aimed to administer the survey to 1000 participants, 500 per coverage area, we interviewed 1,194 individuals overall.

### Implementation

We contracted an independent polling company to implement the study. Interviewers possessed at least a secondary education and had extensive experience implementing surveys. We led a training session to educate interviewers on the study’s objectives and technical topics related to vaccination.

Unvaccinated children were defined as having received none of the vaccines in the national schedule. Undervaccinated children were defined as those missing one or more vaccines [7].

Participants were contacted in their homes from 12–18 May 2011. Interviewers administered the questionnaire to participants and recorded answers electronically on Palm Pilot devices. Interviewers did not review children’s vaccination cards. Instead, in order to evaluate the completeness of the immunization schedule, participants were asked whether their children had received the following vaccines: BCG, Influenza, MMR, OPV, pentavalent, the tetanus-diphtheria vaccine for adult women, and the first and second boosters of DPT and OPV. Responses were then compared to the vaccines that the child should have received per the national schedule.

National officials in Guatemala’s NIP considered the study to be a public health operational evaluation. As such, the MSPAS was not required to seek ethical approval to conduct the study. However, interviewers attained informed verbal consent from all participants. While participants provided their telephone numbers, they did not provide their names or addresses, and the polling company strictly guarded all personal information to guarantee the anonymity of all participants.

Interviews lasted an average of 25 minutes and were conducted from 10:00 a.m. - 7:00 p.m. on weekdays and from 9:00 a.m. - 6:00 p.m. on weekends. Participants were free to terminate the interview at any time. To ensure quality and completeness, a supervisor validated all surveys on the day of completion. If information was incomplete or inconsistent, supervisors attempted to contact participants using the phone numbers provided. Questionnaires were excluded from the analysis if the participant could not be reached or if the information supplied was considered unreliable.

### Questionnaire

Based on a review of the literature on the epidemiology of undervaccinated children, we developed a preliminary version of the questionnaire. The questionnaire was intended to measure both factors facilitating immunization (e.g., short wait times) and those inhibiting immunization (e.g., poor quality service) [6]. We defined risk factors for undervaccination as those factors for which correlation but not causality could be established (e.g. age of the child’s caregiver). Conversely, barriers to immunization were defined as the reasons participants offered for why their children had not or might not be vaccinated. Several rounds of technical revisions were made to ensure that the survey was written clearly and measured all target variables. Prior to implementation, we tested the surveying tools in the field to guarantee that potential participants understood all questions and answer choices.

The final version of the questionnaire contained 85 questions and sub-questions divided into categories pertaining to demographic and socioeconomic data, knowledge of vaccination and awareness of communication strategies, parental attitudes and perceptions concerning vaccination, parental vaccination practices and preferences, indicators of the country’s immunization services, and the children’s immunization history. If a participant had multiple children aged 0–5 years, interviewers repeated questions for individual children as appropriate. Five types of questions were used: recall (participant provides answer without choices); dichotomous (participant chooses one of two options); level of measurement (participant indicates preference of choices); aided response (participant unable to answer and interviewer provides assistance); and multiple response (participant may select multiple options to support response) [8]. To aid comprehension of some responses, the survey contained visual indicators, such as smiley faces, corresponding to different levels of agreement.

### Data analysis

We performed data analyses using version 14 of Statistical Package for the Social Sciences Software (SPSS). Risks ratios (RR) with p values (Yates’ corrected when appropriate) and 95% confidence intervals (CI) were calculated using version 12.5 of MedCalc, an online statistical software [9].

## Results

The study’s 1194 participants had a total of 1593 children aged 0–5 years. Of the participants, 85.1% were housewives, 70.3% belonged to households with monthly incomes < $256 United States Dollars (USD), and 63.9% possessed no more than a primary education (Table 1). Approximately 15% of eligible participants contacted declined participation.

**Table 1:** Characteristics of participants by coverage area: 10 departments of Guatemala, May 2011

**Characteristic**	**Low-coverage departments (n = 632) no. (%)**	**High-coverage departments (n = 562) no. (%)**
***Sex***		
Male	68 (10.8)	71 (12.6)
Female	564 (89.2)	491 (87.4)
***Age***		
15-19	52 (8.2)	47 (8.4)
20-24	151 (23.9)	154 (27.4)
25-39	334 (52.9)	290 (51.6)
>39	95 (15.0)	71 (12.6)
***Education***		
None	100 (15.8)	87 (15.5)
Primary or less	287 (45.4)	289 (51.4)
Secondary^a^	228 (36.1)	171 (30.4)
Post-secondary	17 (2.7)	15 (2.7)
***Occupation***		
Housewife	531 (84.0)	485 (86.3)
Day-laborers	13 (2.1)	11 (2.0)
Professionals	18 (2.8)	20 (3.6)
Business-related	26 (4.1)	16 (2.8)
Other	44 (7.0)	30 (5.3)
***Marital Status***		
Single	96 (15.2)	74 (13.2)
Married	316 (50.0)	296 (52.7)
In relationship	201 (31.8)	179 (31.9)
Divorced	5 (0.8)	3 (0.5)
Other	14 (2.2)	10 (1.7)
***Monthly income***		
<$128	192 (30.4)	247 (44.0)
$128-256	228 (36.1)	172 (30.6)
$256-384	87 (13.8)	67 (11.9)
>$384	48 (7.5)	12 (2.1)
No response/I don’t know	77 (12.2)	64 (11.4)
***Children aged 0–5 years***		
1	449 (71.0)	421 (74.9)
2	135 (21.4)	117 (20.8)
3	38 (6.0)	22 (3.9)
4 or more	10 (1.6)	2 (0.4)
***Age of child (years)***^***b***^		
0-1 (n = 247)	128 (14.7)	119 (16.4)
1 (n = 296)	153 (17.6)	143 (19.7)
2 (n = 276)	150 (17.3)	126 (17.4)
3 (n = 275)	167 (19.2)	108 (14.9)
4 (n = 287)	142 (16.4)	145 (20.0)
5 (n = 212)	128 (14.8)	84 (11.6)
Total (n = 1593)	868 (100.0)	725 (100.0)

^a^The term “secondary” includes participants with any level of post-primary education.

^b^While the age of each participant’s child was recorded, sex was not.

### Knowledge

Of participants with secondary or post-secondary education, 58.0% (n = 250/431) said they knew “a lot” or “something” about vaccination. This proportion was lower for participants with no more than a primary education (44.4%, n = 339/763, p < 0.001). Similarly, 39.1% (n = 158/404) of caregivers aged <25 years reported significant knowledge, compared to 54.6% (n = 431/790) of those aged >25 years (p < 0.001).

Participants generally recognized vaccines in the national schedule. Polio vaccine was mentioned most frequently (95.9%), while pentavalent and MMR vaccines were also widely recognized (88.4% and 88.0%, respectively). Among vaccines in the national schedule, the tetanus-diphtheria vaccine for adult women was least well known (77.2%).

### Attitudes

A total of 1138 (95.3%) participants considered vaccination “very necessary,” 1005 (84.2%) considered it “very important,” and 1152 (96.5%) said “vaccination protects people against diseases” (Table 2). The proportion of participants who considered vaccination to be “very important” was higher in low-coverage areas (90.0%) than in high-coverage areas (77.6%, p < 0.001). Despite participants’ strong belief in immunization, 1073 (89.9%) indicated that they lacked at least some information on the necessity of vaccination (Table 3). Although more participants in low-coverage areas indicated they lacked this information (90.8% vs. 88.8%), the difference was not statistically significant (p = 0.245). Reported advantages of vaccination centered on the child’s welfare. Disadvantages included “adverse events” (36.2%), “the child’s pain” (27.6%), and “cost” (2.8%).

**Table 2:** Factors facilitating vaccination by coverage area: 10 departments of Guatemala, May 2011

**Factors facilitating vaccination (% of respondents agreeing with statement)**	**Total (n = 1194) no. (%)**	**Department type**		
		**Low-coverage (n = 632) no. (%)**	**High-coverage (n = 562) no. (%)**	**Chi-square (p value)**^**a**^
***Structural***				
The cost of vaccines is NOT a disadvantage	1161 (97.2)	612 (96.8)	548 (97.5)	0.485
There is a place nearby where I can vaccinate my child	987 (82.7)	518 (82.0)	469 (83.5)	0.497
***Parental attitudes***				
Vaccination is “very important”	1005 (84.2)	569 (90.0)	436 (77.6)	**<0.001**
Vaccination is “important” or “very important”	1191 (99.8)	630 (99.7)	561 (99.8)	0.663
Vaccination is “very necessary”^b^	1138 (95.3)	606 (95.9)	532 (94.7)	0.318
Vaccines protect against diseases^b^	1152 (96.5)	612 (96.9)	540 (96.0)	0.483
***Parental practice***				
All my children have vaccination cards (n = 1593)^c^	1532 (96.2)	831 (95.8)	701 (96.7)	0.324
I am aware that health centers offer immunization services	1133 (94.9)	599 (94.8)	534 (95.0)	0.851
I decide to vaccinate my child when a healthcare professional tells me to do so	692 (58.0)	400 (63.3)	292 (51.9)	**<0.001**
I decide to vaccinate my child when he or she is sick	145 (12.1)	60 (9.5)	85 (15.1)	**0.003**
***Quality of service***				
I typically wait <1 hour to vaccinate my child^d^	687 (58.2)	331 (52.9)	356 (64.1)	**<0.001**
I have ALWAYS been able to vaccinate my child at a health center	1015 (85.0)	530 (83.9)	485 (86.3)	0.239
Service is “good ” or “very good”^d^	831 (70.4)	425 (67.9)	406 (73.2)	**0.048**
Service is “average”^d^	302 (25.6)	172 (27.5)	130 (23.4)	0.111

^a^Chi-square tests were performed to compare characteristics in high- and low-coverage areas. Statistically significant values (p < 0.05) are bolded.

^b^Totals include respondents who agreed or strongly agreed with statement; respondents who disagreed or somewhat agreed are excluded.

^c^Totals include all children studied (n = 1593). Percentages are based on number of children in low- and high-coverage areas (868 and 725, respectively).

^d^Thirteen participants did not respond and were excluded. Percentages are based on the number of participants in low- and high-coverage areas (626 and 555, respectively).

**Table 3:** Factors inhibiting vaccination by coverage area: 10 departments of Guatemala, May 2011

**Factors inhibiting vaccination (% of participants agreeing with statement)**	**Total (n = 1194) no. (%)**	**Department type**		
		**Low-coverage (n = 632) no. (%)**	**High-coverage (n = 562) no. (%)**	**Chi-square (p value)**^**a**^
***Structural***				
I have been denied service due to the lack of vaccine	78 (6.5)	46 (7.3)	32 (5.7)	0.269
I have been denied service due to the lack of medical personnel or attention	39 (3.3)	22 (3.5)	17 (3.0)	0.780
I live >3 km from a vaccination site	323 (27.1)	188 (29.7)	135 (24.0)	**0.026**
***Quality of service***				
I typically wait >2 hours to vaccinate my child^b^	164 (13.7)	102 (16.3)	60 (10.8)	**0.006**
Upon having my child vaccinated, I receive some form of advice from health workers^b^	861 (72.9)	464 (74.1)	397 (71.5)	0.318
Health workers inform me of the risk of adverse events^b^	560 (47.4)	303 (48.4)	257 (46.3)	0.471
Health workers tell me my child’s next vaccination appointment^b^	382 (32.3)	207 (33.1)	175 (31.5)	0.573
Health workers tell me how many doses are required for vaccines to be effective^b^	581 (49.2)	328 (52.4)	253 (45.6)	**0.019**
***Communication***				
I lack information on why vaccination is necessary^c^	1073 (89.9)	574 (90.8)	499 (88.8)	0.245
I do NOT know or believe that vaccination services are available year-round^d^	401 (33.6)	180 (28.5)	221 (39.4)	**<0.001**
I have NOT heard information about immunization campaigns	616 (51.6)	305 (48.3)	311 (55.3)	**0.015**

^a^Chi-square tests were performed to compare characteristics in high- and low-coverage areas. Statistically significant values (p < 0.05) are bolded.

^b^Thirteen participants did not respond and were excluded. Percentages are based on the number of participants in low- and high-coverage areas (626 and 555, respectively).

^c^Total includes respondents who agreed, somewhat agreed, and strongly agreed with the statement; respondents who disagreed or did not respond were excluded.

^d^Total includes respondents who answered the question “no” as well as those who did not respond or said they did not know.

### Practices

Participants indicated that 1565 (98.2%) children aged 0–5 years had received at least one vaccine. In comparing the child’s age with parental recall on vaccines administered, we determined that 1106 children (69.4%) had complete vaccination schedules, 458 (28.8%) had incomplete schedules, and 29 (1.8%) had no history of vaccination.

Proportions of children with complete schedules varied by age and antigen. Based on maternal recall data, children aged <2 years were more likely to have complete schedules than those aged 2–5 years (86.9% vs. 60.4%). Regardless of coverage area, differences between younger and older children were found to be statistically significant (Table 4). However, children in low-coverage areas were not found to be at a higher risk for undervaccination (RR = 0.93, p = 0.31). According to participants, 94.0% of eligible children had received BCG at birth. Similarly, 93.3% had received at least one dose of the pentavalent, polio, and rotavirus vaccines administered before age <1 year. Fewer parents reported vaccinating their children with MMR at age 1 year (88.8%) and with the required polio and DPT boosters at ages 18 months and 4 years (90.3% and 76.2%, respectively).

**Table 4:** Risk factors for incomplete vaccination schedules by coverage level: 10 departments of Guatemala, May 2011

**Characteristic**	**Low-coverage departments (n = 868)**				**High-coverage departments (n = 725)**			
	**Immunization schedule**		**Univariate risk factor analysis**^**a**^		**Immunization schedule**		**Univariate risk factor analysis**	
	**Incomplete (n = 256) no. (%)**	**Complete (n = 612) no. (%)**	**Prevalence ratio (95% confidence interval)**	**P**	**Incomplete (n = 231) no. (%)**	**Complete (n = 494) no. (%)**	**Prevalence ratio (95% confidence interval)**	**P**
***Age of caregiver***								
15-19	19 (31.7)	41 (68.3)	1.11 (0.74, 1.65)	0.62	14 (26.4)	39 (73.6)	0.87 (0.54, 1.39)	0.55
20-24	54 (25.8)	155 (74.2)	0.90 (0.69, 1.18)	0.46	64 (32.5)	133 (67.5)	1.06 (0.83, 1.37)	0.63
25-39	134 (28.6)	334 (71.4)	**Reference**		120 (30.5)	273 (69.5)	**Reference**	
>39	49 (37.4)	82 (62.6)	1.31 (1.00, 1.70)	**0.047**	33 (40.2)	49 (59.8)	1.32 (0.97, 1.78)	0.07
***Education***								
None	52 (34.4)	99 (65.6)	1.49 (1.10, 2.00)	**0.010**	47 (43.2)	62 (56.8)	1.72 (1.26, 2.34)	**0.0007**
Primary or less	134 (32.3)	281 (67.7)	1.39 (1.09, 1.79)	**0.009**	127 (32.6)	262 (67.4)	1.30 (1.00, 1.70)	**0.05**
More than primary	70 (23.1)	232 (76.8)	**Reference**		57 (25.1)	170 (74.9)	**Reference**	
***Occupation***								
Other	35 (25.9)	100 (74.1)	0.86 (0.63, 1.17)	0.33	31 (32.3)	65 (67.7)	1.02 (0.74, 1.39)	0.92
Housewife	221 (30.2)	512 (69.8)	**Reference**		200 (31.8)	429 (68.2)	**Reference**	
***Marital status***^***b***^								
Single^c^	54 (36.8)	91 (63.2)	1.32 (1.02, 1.71)	**0.0347**	42 (39.6)	64 (60.4)	1.36 (1.03, 1.80)	**0.03**
Married	124 (28.2)	316 (71.8)	**Reference**		113 (29.1)	275 (70.9)	**Reference**	
Civil union	77 (27.6)	202 (72.4)	0.98 (0.77, 1.25)	0.86	75 (32.9)	153 (67.1)	1.13 (0.89, 1.44)	0.32
***No. of children 0-5***^***d***^								
1st child	191 (30.2)	441 (69.8)	**Reference**		195 (34.7)	367 (65.3)	**Reference**	
2nd child	48 (26.5)	133 (73.5)	0.88 (0.67, 1.15)	0.34	29 (20.7)	111 (79.3)	0.60 (0.42, 0.84)	**0.0032**
3rd child or more	17 (30.9)	38 (69.1)	1.02 (0.68, 1.55)	0.91	7 (30.4)	16 (69.6)	0.88 (0.47, 1.64)	0.68
***Age of children***								
0-1	17 (13.4)	110 (86.6)	**Reference**		20 (16.8)	99 (83.2)	**Reference**	
1	18 (11.7)	136 (88.3)	0.87 (0.47, 1.62)	0.67	16 (11.2)	127 (88.8)	0.67 (0.36, 1.23)	0.19
2	71 (47.3)	79 (52.7)	3.54 (2.20, 5.68)	**<0.0001**	48 (38.1)	78 (61.9)	2.27 (1.43, 3.58)	**0.0005**
3	42 (25.1)	125 (74.9)	1.88 (1.12, 3.14)	**0.016**	39 (36.1)	69 (63.9)	2.15 (1.34, 3.44)	**0.0015**
4	72 (50.7)	70 (49.3)	3.79 (2.36, 6.07)	**<0.0001**	80 (55.2)	65 (44.8)	3.28 (2.14, 5.03)	**<0.0001**
5	36 (28.1)	92 (71.9)	2.10 (1.25, 3.54)	**0.0053**	28 (33.3)	56 (66.7)	1.98 (1.20, 3.27)	**0.0074**

^a^A univariate risk factor analysis was performed to determine factors associated with undervaccination in high- and low-coverage areas using Confidence Intervals of 95%. Groups with incomplete schedules were placed in the “positive outcome” column, while those with complete schedules were placed in the “negative outcome” column. Statistically significant values (p < 0.05) are bolded.

^b^Under this category, seven cases marked as “other” were excluded.

^c^The category “single” includes participants who indicated being single, divorced, or widowed.

^d^Participants were asked if they had multiple children aged 0–5 years. Categories correspond to the birth order of only those children aged 0–5 years.

Of the 1593 children studied, 1532 (96.2%) had vaccination cards, according to respondents. Mothers reported being responsible for vaccinating their child in most cases (n = 1118, 93.6%). Participants indicated that recommendations of pediatricians and health center staff influence their decisions to vaccinate, with 692 (58.0%) reporting that they vaccinate their child following such a recommendation. In low-coverage areas, the role of the pediatrician and health center staff appears to play a greater role in influencing parental decisions (63.3% vs. 51.9%, p < 0.001). In high-coverage areas, more caregivers reported vaccinating their children when they become sick (15.1% vs. 9.5%, p = 0.003).

### Preferences

Participants preferred to vaccinate their children on business days (78.5%) and for vaccination campaigns to take place in the morning (87.7%). Most participants (94.9%) identified health centers as possible vaccination sites, but 26.0% cited public hospitals, 7.0% the Social Security Institute (IGSS), and 6.5% private hospitals. When asked to identify the site of their child’s last vaccination, 77.1% of participants said health centers, 7.8% said the Social Security Institute (IGSS), and 2.2% said vaccination campaigns. Participants in low-coverage areas reported a greater likelihood to vaccinate their children at the IGSS (12.6% vs. 2.3%, p < 0.001), while participants in high-coverage areas disproportionately reported vaccinating their children at health centers (84.5% vs. 70.4%, p < 0.001).

When asked to identify the most important feature of immunization services, 309 (25.9%) chose vaccine availability and 247 (20.7%) chose the health center’s proximity to their home. Notably, 1081 (90.5%) participants affirmed that the “free cost of vaccines” is an important consideration. But, only 41 (3.4%) said they might be unable to vaccinate their children due to cost, be it the direct cost of vaccination or indirect costs associated with accessing services (e.g., transportation or lost wages).

### User-satisfaction

Overall*,* 831 (70.4%) participants rated immunization services as “good” or “very good,” 302 (25.6%) as “average,” and 47 (4.0%) as “bad” or “very bad.” Seventy-three percent of participants in high-coverage areas considered service “good” or “very good,” compared to only 67.8% in low-coverage areas (p = 0.048). “Free vaccines” (n = 256, 30.8%) and the “ability of medical personnel to provide immediate attention” (n = 531, 63.9%) were the principal reasons given for a positive evaluation. “Poor quality service” (n = 20, 41.7%) and “rude treatment” (n = 31, 64.6%) were the main reasons given for a negative evaluation.

### Quality of service and factors inhibiting immunization

Of 1194 participants, 987 (82.7%) said that a vaccination center was located close to their homes. This response did not differ by coverage area, but participants in low-coverage areas estimated that they needed to travel an average of 6.8 km to health centers, while those in high-coverage areas reported traveling an average of 3.3 km. Compared to high-coverage areas, more parents in low-coverage areas reported living >3 km from a vaccination site (p = 0.026).

Wait times at health centers varied. The majority of participants said they waited <1 hour to vaccinate their child, but 26.8% waited 1–2 hours and 13.7% waited >2 hours. More residents in low-coverage areas reported wait times >2 hours (16.3% vs. 10.8%, p = 0.006). Most participants reported receiving some form of advice upon having their child immunized (n = 861, 72.9%). Those claiming to have received no advice (n = 320, 27.1%) generally attributed the lack of communication to the busyness of the medical professional. According to participants, important topics such as the risk of adverse events (47.4%), the child’s next vaccination appointment (32.3%), and the number of doses required for vaccine effectiveness (49.2%) were inconsistently communicated to parents and guardians.

When asked to identify reasons why they might be unable to vaccinate their children, participants mentioned the “sickness of the child” (26.5%), “lack of time” (20.2%), “lack of vaccines” (10.1%) and “lack of vaccinating personnel” in health centers (7.6%), “high cost” (3.4%), and “distance to health centers” (3.3%). A total of 179 (15.0%) participants reported having been denied immunization services. Reasons included “lack of vaccines” (n = 78), “lack of medical personnel or service” (n = 39), and “excessive wait times” (n = 9), among others. While the proportion of respondents who were denied service was greater in low-coverage than in high-coverage areas (16.1% vs. 13.7%), the difference was not statistically significant (p = 0.24).

In low-coverage areas, children of less educated (no education: RR = 1.49, p = 0.01; primary or less: 1.39, p = 0.009), older (aged >39 years: RR =1.31, p = 0.05), and single (RR = 1.32, p = 0.03) parents were more likely to have incomplete immunization schedules. Factors related to undervaccination in high-coverage areas included the caregiver’s lack of education (none: RR = 1.72, p = 0.0007; primary or less: RR = 1.30, p = 0.05), single marital status (RR = 1.36, p = 0.03) and the child’s birth order (second: RR = 0.60, p = 0.003). Children aged ≥2 years in both coverage areas had higher relative risks of undervaccination.

### Communication strategies

**Figure 2:**
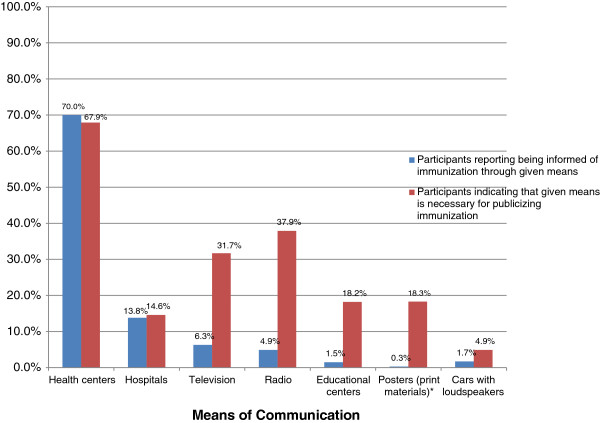
**Means of communication, practices vs. preferences: 10 departments of Guatemala, May 2011.** *Participants were not asked specifically whether they had received information on vaccination from posters. Rather, they were asked if they had received information from printed materials (0.3%). However, 18.3% of participants indicated that posters should be used to publicize immunization.

Participants in low-coverage areas showed greater awareness of vaccination than those in high-coverage areas. We found differences between how participants became aware of services and the means by which they believed information on vaccination should be publicized (Figure 2). Only 18 (1.5%) participants became aware of vaccination through schools, but 217 (18.2%) believed vaccination should be publicized in educational centers. Participants were most likely to recommend educational sessions in the community (n = 423, 35.4%) as the best way for the government to promote vaccination.

A total of 587 (48.4%) participants said they had recently heard of a vaccination campaign and 683 (57.2%) said they took advantage of campaigns. Although 693 (58.0%) participants said health centers were the best place to implement campaigns, 432 (36.2%) suggested that schools were the better setting.

## Discussion

The present study is the first analysis on the causes of undervaccination in Guatemala since 1990 [10]. We found that Guatemala’s immunization program is well regarded and that the country enjoys a strong vaccination culture. The low cost of vaccines (free in public health facilities) and the population’s belief in the importance of immunization should facilitate the program’s continued success. To address new challenges, the NIP must face immunization barriers that contribute to substandard coverage rates. While risk factors associated with undervaccination were similar between coverage areas, barriers varied. In low-coverage departments, barriers related to structural issues: vaccine shortages, excessive wait times, and long distances to health centers. In high-coverage departments, participants reported being less aware of immunization services. Throughout Guatemala, the reported failure of health workers to provide information on vaccines administered, next appointments, and adverse events may result in many parents who leave health centers confused and uncertain of when to next vaccinate their children.

Several limitations to this study should be acknowledged. Though the 10 departments surveyed account for nearly half of Guatemala’s population, the study is not representative of the country. The non-response rate was high and no data was collected on the reasons for refusing participation. More significantly, participants provided responses based on memory and interviewers did not verify these answers against the child’s vaccination card. Results are thus subject to respondent and recall bias, which may be more important for caregivers of children aged ≥ 2 years. For example, the finding that older children had greater relative risks of undervaccination was likely affected by parental lapses in memory, even if it is also related to the fact that the number of vaccines needed for a child to be fully vaccinated increases with age. Additionally, parents may not remember having been told of their child’s next vaccination appointment, resulting in an underestimation of service quality. Conversely, the proportion of children with complete immunization schedules is likely overestimated. Because second- and third-dose data for OPV, DPT, and rotavirus vaccines were not collected, the true proportion of children in this study with incomplete schedules is unknown.

The survey was administered only in Spanish and did not include a question on race or ethnicity, thus preventing the identification of risk factors associated with these criteria. Previous health studies have demonstrated that indigenous populations in Guatemala have greater difficulty accessing health services and highlight the importance of further research evaluating the specific challenges of these communities [11-13]. Finally, this study did not provide information on causes of vaccine shortages, the reasons that health workers provide incomplete service, the specific information on vaccination that parents consider lacking, and other aspects of the NIP that national officials may need to design the most effective interventions.

Despite these limitations, the data collected provides a baseline to evaluate interventions and a conservative calculation of relative risks for undervaccination. Similarities between our results and those of other health studies, including the Demographic and Health Surveys (DHS), suggest that our findings are reliable [11]. Coverage rates determined in this study by parental recall are similar to reported rates, particularly for one-dose vaccines (e.g., 88% MMR1 reported in 2011 vs. 88.8% based on parental memory) [2]. Furthermore, our findings are consistent with the few studies on undervaccinated children in Guatemala. In 1990, false contraindications, lack of vaccination cards, and poor quality service were identified as contributors to low coverage rates [10]. Since then, many challenges have been met—e.g., most Guatemalans now have vaccination cards—but others remain. Below, we propose potential solutions to these problems.

### Design and implement targeted communication strategies to increase demand of services

Guatemalans are committed to vaccination, but 89.9% said they lacked at least “some information” on the subject. In particular, participants aged <25 years and those lacking a secondary education considered themselves less knowledgeable, and those aged >39 years were more likely to have children with incomplete schedules. Associations between undervaccination and parental education and marital status and the child’s age are consistent with previous studies and reaffirm the need for communication strategies targeting high-risk populations [10,11].

Notably, most participants were not aware of a recent vaccination campaign and only 26 (2.2%) reported last vaccinating their child at a campaign. While the underlying causes of this finding are poorly understood, the fact that this study was conducted three weeks after Vaccination Week in the Americas (VWA) suggests that VWA in particular and vaccination campaigns more generally must be better publicized. VWA offers a valuable opportunity to promote immunization both among the public and within the government.

### Hold educational sessions to increase the public’s knowledge of vaccination

Though 98.2% of parents and guardians said their children were vaccinated, at least 30.6% had children with incomplete schedules. Children aged >1 year were more likely to be undervaccinated, with many not having received MMR or the first and second boosters of OPV and DPT. Communication strategies should emphasize that a child i*s only fully vaccinated* when she has received all doses of all required vaccines. Parents must also be reminded that possessing vaccination cards is not sufficient—the cards must be used. In addition to incorporating these messages into communication strategies, educational sessions should be held at the local level. Participants repeatedly indicated that community-level workshops are the best way to increase user awareness and knowledge of vaccination.

### Ensure availability of vaccines and medical personnel

Nearly one in ten participants reported having been denied service due to the lack of vaccines or medical personnel. Possible causes include inaccurate forecasting of vaccine demand, cold chain deficiencies, and poor organization resulting from the high turnover of health center staff. At the local level, program managers must determine the underlying causes of vaccine and personnel shortages. At the national level, policies should be implemented to minimize the turnover of personnel. A long-term solution may be to establish a recognized career path in civil service to strengthen the government’s administrative capacity [14]. For the present, the NIP might consider implementing catch-up activities targeting children aged 2–5 years to reduce the number of children with delayed immunization schedules. Lastly, health centers enjoy wide recognition as the primary sites for immunization, but opportunities may exist elsewhere. The NIP should investigate whether schools, or other community centers, can assume greater roles in promoting and providing immunization services.

### Improve service quality

Many participants (27.1%) reported receiving no advice from healthcare professionals upon vaccinating their child. While the number of children who fail to receive subsequent vaccines due to incomplete care is unknown, it may be substantial. Since 32.3% of parents claimed to have been told when next to vaccinate their child, more than two-thirds of children may run an increased risk of missing their next appointment. What is more, the failure of medical personnel to provide basic information to caregiver likely contributes to the perception that information on immunization is lacking. In light of these challenges, the NIP should increase the training and supervision of health workers who offer immunization services, particularly in low-coverage departments.

While the multiple demands placed on health workers, especially in underserved areas, contributes to the incomplete care reported by many users, health workers should be required to provide parents a “next appointment” card. The paperwork accompanying the child’s visit might also include a short checklist reminding health workers to 1) explain the risk of reactions to vaccines and what to do when such reactions occur; 2) indicate the number of vaccine doses required for immunity; and 3) review the child’s card with the parent, indicating the next date of vaccination. Simple checklists are increasingly used in clinical settings to prevent errors and increase quality of care [15,16].

### Lessons learned

This study offers lessons relevant to future immunization surveys and regional efforts to develop a standardized methodology for detecting missed opportunities for vaccination (MOV). Foremost among these is the value of contracting an external evaluator to implement the study. Polling companies offer surveying expertise and independent perspective that can prove helpful in designing interventions. Regarding survey design, small changes in language may have large implications in data analysis and permit countries to monitor progress over time. In this respect, the questionnaire used in Guatemala might have been improved. Instead of asking participants if they *had ever been* denied vaccination services, participants might have been asked if they were denied service *during their last visit to a health center*. Lastly, the use of Palm Pilots in this study facilitated data collection, prevented recording and coding errors, and allowed for preliminary results to be promptly delivered to national authorities. Future studies should consider using this and other technologies.

## Conclusion

Guatemala enjoys a strong vaccination culture and users are generally satisfied with national services. But several factors explain why homogenous coverage of ≥95% has not been achieved. These relate primarily to structural and communication issues, such as the lack of vaccines and effective marketing strategies, rather than to a population that does not value vaccination. To maintain its achievements and address new challenges, the NIP should increase the training and supervision of health workers who offer immunization services. Additional recommendations include improving vaccine forecasting and supply to avoid stockouts, holding educational sessions to increase public awareness and knowledge of vaccination, and implementing catch-up activities targeting children aged 2–5 years to reduce the number of children with delayed immunization schedules.

## Abbreviations

NIP: National Immunization Program; CRS: Congenital rubella syndrome; DPT3: Third-dose coverage of diphtheria, tetanus, and pertussis vaccine; BCG: Bacillus Calmette-Guerin; MMR: Measles-rubella-mumps containing vaccine; OPV3: Third dose of oral polio vaccine; MSPAS in Spanish: Ministry of Health and Social Protection; IGSS in Spanish: Guatemalan Social Security Institute; PAHO: Pan American Health Organization; INE in Spanish: National Institute of Statistics; PSU: Primary sampling unit; SS: Sample space; SPSS: Statistical Package for the Social Sciences Software; RR: Risks ratios; CI: Confidence interval; USD: United States dollars; VWA: Vaccination Week in the Americas; DHS: Demographic and health surveys.

## Competing interests

The authors declare that they have no competing interests.

## Authors’ contributions

LB designed the study and contributed to the development of the surveying tools. SPT, MV, and PB participated in the data analysis. SPT drafted the manuscript with contributions from LB, MV, PB, and MCD-H. All authors read and approved the manuscript.

## Authors’ information

LB is the manager of Guatemala’s National Immunization Program. SPT worked for PAHO from 2008–2013, mostly recently as a consultant on immunization. He is now a student at the Vanderbilt School of Medicine in Nashville, Tennessee. PBA is a Peruvian national with a master’s degree in public health. She has 11 years of experience in public health and works as a technical officer with PAHO’s Immunization Project. A Colombian physician, MVG served as the manager of Colombia’s immunization program for five years. In 2011, she joined PAHO as a regional immunization advisor. MCD-H is a Chilean physician with a master’s degree in epidemiology. Since 2004, she has served as a regional immunization advisor, overseeing immunization data quality for the organization.

## Pre-publication history

The pre-publication history for this paper can be accessed here:

http://www.biomedcentral.com/1471-2458/14/231/prepub
